# Linking Multi-Omics to Wheat Resistance Types to *Fusarium* Head Blight to Reveal the Underlying Mechanisms

**DOI:** 10.3390/ijms23042280

**Published:** 2022-02-18

**Authors:** Fan Wu, Yao Zhou, Yingying Shen, Zhengxi Sun, Lei Li, Tao Li

**Affiliations:** Key Laboratory of Plant Functional Genomics of the Ministry of Education/Jiangsu Key Laboratory of Crop Genomics and Molecular Breeding/Collaborative Innovation of Modern Crops and Food Crops in Jiangsu/Jiangsu Key Laboratory of Crop Genetics and Physiology, College of Agriculture, Yangzhou University, Yangzhou 225009, China; fanwu18762303998@163.com (F.W.); yaozhou1231@163.com (Y.Z.); yingyingshen123456@163.com (Y.S.); zhengxisun@yzu.edu.cn (Z.S.); lilei@yzu.edu.cn (L.L.)

**Keywords:** *Fusarium* head blight, multi-omics, resistance mechanism, resistance types

## Abstract

*Fusarium* head blight (FHB) caused by *Fusarium graminearum* is a worldwide disease which has destructive effects on wheat production, resulting in severe yield reduction and quality deterioration, while FHB-infected wheat grains are toxic to people and animals due to accumulation of fungal toxins. Although impressive progress towards understanding host resistance has been achieved, our knowledge of the mechanism underlying host resistance is still quite limited due to the complexity of wheat–pathogen interactions. In recent years, disease epidemics, the resistance germplasms and components, the genetic mechanism of FHB, and disease management and control, etc., have been well reviewed. However, the resistance mechanism of FHB is quite complex with Type I, II to V resistances. In this review, we focus on the potential resistance mechanisms by linking different resistance types to multi-omics and emphasize the pathways or genes that may play significant roles in the different types of resistance. Deciphering the complicated mechanism of FHB resistance types in wheat at the integral levels based on multi-omics may help discover the genes or pathways that are critical for different FHB resistance, which could then be utilized and manipulated to improve FHB resistance in wheat breeding programs by using transgenic approaches, gene editing, or marker assisted selection strategies.

## 1. Introduction

*Fusarium* head blight (FHB), mainly caused by *Fusarium graminearum* (*F. graminearum*), is a destructive wheat (*Triticum aestivum* L.) disease in warm and humid regions worldwide, causing premature spike death or blighting, and substantially reducing grain yield and quality [1]. The disease brings globally devastating economic losses and poses a threat to the health of humans and animals [2]. Schroeder and Christensen first reported two types of resistance: resistance to initial pathogen penetration (Type I) and resistance to the spread of disease within a spike (Type II) [3]. Based on different host reactions to FHB infection, other resistance mechanisms have also been proposed: Type III resistance to mycotoxin accumulation, Type IV resistance to kernel infection, and Type V resistance for tolerance to yield loss [4,5]. Recently, Gong et al. [6] proposed the merger of Type V into type IV resistance because of their similarity in nature to simplify the FHB resistance types, and to use the reduction in thousand kernel weight (TKW) under the premise of the same disease severity as an indicator for Type IV resistance. Among these types, Type II and Type III have been studied more extensively because of their significance in association with yield loss and with the degree of grain toxin contamination directly related to food safety [7].

FHB resistance is a quantitative trait and controlled by several quantitative trait loci (QTL) [8]. In recent years, hundreds of QTL have been reported [9]. Up to now, seven QTL conferring resistance to FHB have been formally designated: *Fhb1* on chromosome 3BS [10] and *Fhb2* on chromosome 6BS both from ‘Sumai3’ [11], *Fhb3* on chromosome 7AS from *Leymus racemosus* [12], *Fhb4* on chromosomes 4B [13], *Fhb5* on chromosomes 5A [14] from ‘Wangshuibai’, *Fhb6* on chromosome 1A from *1Ets#1S of Elymus tsukushiensis* [15], and *Fhb7* on chromosome 7E from *Thinopyrum elongatum* [16]. Among these QTL, *Fhb1* has the largest effect on FHB resistance and is more stable across genetic backgrounds [17]. Only two QTL (*Fhb1* and *Fhb7*) have been claimed to being cloned, however, their functions remain controversial [16,18,19,20,21,22], probably due to the complexity of huge hexaploid wheat genome, phenotyping methods applied and phenotypic accuracy, and (or) complicated interaction of wheat–*Fusarium* species.

Plant resistance to pathogen attack is regulated by a complex gene network. Breeding wheat cultivars with a high level of resistance to FHB is one of the most economical, environmentally safe, and effective means of disease control [1]. Up to date, only Type II resistance has been well characterized and widely utilized in breeding research, as it is more stable and easier to assess [8]. It is difficult to breed FHB-resistant wheat cultivars because of the complex wheat genome, quantitative inheritance, variability of phenotype, and the unclear resistance mechanism in host-pathogen interaction. Thus, wheat breeding programs have been limited by a lack of effective resistance genes [23]. Notably, widely popular ‘OMICs’ approaches have driven many significant advances in biomedical research, enabling researchers to generate huge datasets at multiple levels of biological organization over the past two decades [24]. Omics, including genomics, proteomics, metabolomics, transcriptomics and so on, give us powerful ways and diverse dimensions to understand host resistance to FHB with several good reviews on disease resistance or detoxification [25,26]. However, the association of different types of resistance with omics has seldom been published. In this paper, we summarized and reviewed omics data collected spatio-temporally in wheat during wheat-FHB interaction and addressed the defense mechanisms of different resistance types by integrating genomics (here we refer to QTL), transcriptome, proteome, and metabolome-associated reports. We focused on general host responses to FHB in different resistance types rather than emphasizing case-specific host-pathogen interactions, and to identify resistance-associated genes or pathways that are critical for different FHB resistance types. Analyzing the mechanism of different resistance types would greatly help understand the interaction of different resistance types and discover candidate genes to breed cultivars with better FHB resistance.

## 2. Type I Resistance

Type I resistance is defined as resistance to pathogen penetration at the initial stage of pathogen infection of the host and is traditionally determined by spraying a spore suspension onto flowering spikes and measuring disease incidence (percentage of ears with disease symptoms). Incidence is about host escape of pathogen challenge, therefore it may be irrelevant to host resistance since there is no immune response of the host to the pathogen due to the hemibiotrophic nature of the *Fusarium* species complex [27]. Here we will not address whether incidence is suitable for phenotyping Type I resistance. Disease severity assessed under spray-inoculation experiments is used as a measure of overall FHB resistance because of the mixture of Type I and Type II resistance [9]. Therefore, Type I resistance and Type II resistance cannot be well distinguished by spray inoculation due to the fact that the secondary infections cannot be separated from the primary infection. Macroconidia of pathogen germinated on the surface of all hosts they contacted from 6 h to 12 h after inoculation but do not immediately infect host tissues. A penetration peg, which arises from an infection hypha, invades host tissue by penetrating the inner layers of the host cell wall by 36 hpi [28]. Based on this widely acknowledged observation, we consider that hypha does not penetrate host tissue within 36 hpi and all the omics analyses conducted before this time point, regardless of inoculation methods, are related to Type I resistance as ‘early events’ to distinguish it from Type II resistance.

### 2.1. QTL for Type I Resistance

Type I resistance is considered difficult to assess and fewer reports have been published on Type I resistance QTL [29]. Several QTL specifically associated with resistance to infection such as *Fhb4* and *Fhb5* from ‘Wangshuibai’ [30], *Qfhi.nau-2D* from ‘Y158’ [31], a stable QTL on chromosome 3A from ‘Frontana’ [32], seven QTL on chromosome arms 2DS, 3AS, 3BS, 3BSc (centromeric), 4DL, 5AS, and 6BS from the ‘Sumai3′-derived line ‘DH181’ [33], and a major QTL on the short arm of chromosome 4A [34] have also been reported. These QTL have great effects on resistance expressions.

### 2.2. The Association of Pathways or Enzymes in Early Respiration with Host Responses to FHB

Glycolysis pathway (EMP) is the initial stage of respiration which is a series of reactions that degrade glucose and glycogen to pyruvic acid, accompanied by the formation of ATP. Proteins involved in EMP were identified in *F. graminearum*-inoculated susceptible cultivar ‘Crystal’ and revealed that two pathways including antioxidant and glycolysis were revealed to participate in the direct interaction of *F. graminearum*–wheat [35]. In addition, enolase that functions in EMP has a significant drop in abundance in ‘Wangshuibai’ during *F. graminearum* infection [36]. Several reports showed that most components involved in the EMP pathway were induced or upregulated in susceptible wheat cultivars. Therefore, EMP might be associated with FHB susceptibility in wheat. A recent report also pointed out that succinate dehydrogenase inhibitors (SDHIs) negatively regulated DON biosynthesis, which may be attributed to the inhibitory effects of SDHIs on glycolysis [37].

Tricarboxylic acid cycle (TCA) is a cyclic reaction system composed of a series of enzymatic reactions. TCA is essential for many key reactions and functions in organisms under pathogenic virulence [38]. Biosynthesis of thiamin, which is related to key enzymes in TCA, was increased in response to the pathogen. TCA cycle genes such as aconitases, citrate synthase, and succinate dehydrogenases along with malic enzymes showed greater transcript abundances at 30 hpi compared with 50 hpi in *F. graminearum*-treated lines harboring *Qfhs.ifa-5A* [39]. It is undeniable that TCA cycle genes play important roles in the early stage (within 36 hpi) during *F. graminearum* infection, suggesting that TCA cycle genes may be crucial in Type I resistance to FHB.

Terminal oxidases are enzymes which are at the end of the respiratory chain and can give electrons off the substrate to O_2_ and form H_2_O_2_ or H_2_O including phenol oxidase, flavin oxidoreductase, ascorbate peroxidase and so on. The rapid reactive oxygen species (ROS) production in response to pathogen infection is thought to regulate programmed cell death (PCD) through the establishment of the hypersensitive reaction (HR) [40]. Enhancing or amplifying ROS signals at a later stage will cause PCD. The involvement of antioxidant enzymes in ROS removal is very important in resistance to pathogen infection. FHB-resistant cultivar ‘Vulkan’ showed rapid induction of ascorbate peroxidase (APX) and polyphenol oxidase (PPO) activity in the early stages after infection [41]. Besides, fungal flavin oxidoreductase and APX were detected in FHB-susceptible wheat cultivar ‘Crystal’ with *F. graminearum*-inoculated compared with water-inoculated control [35]. As described above, most substances participating in the terminal oxidase system or respiratory chain were upregulated across resistant and susceptible wheat genotypes and the greater abundance might present in the resistant wheat genotypes rather than the susceptible wheat genotypes, suggesting that the terminal oxidation system may be involved in basal response (The genes or pathways were induced in both resistant and susceptible cultivars with insignificant difference) to FHB at early stage of infection.

It is obvious that TCA cycle genes and terminal oxidases function as early as the initial stage of pathogen infection. The majority of the components involved in the EMP pathway were induced or upregulated in FHB-susceptible wheat genotypes, indicating EMP is connected with FHB susceptibility. In contrast, the higher transcript abundances of genes in the TCA cycle within 36 hpi in resistant cultivars demonstrated their association with FHB resistance, especially Type I resistance. Most substances participating in the terminal oxidase system or respiratory chain may enhance resistance to FHB through early ROS outbreaks at the biotrophic stage. Notably, excessive ROS at the later stages has a strong toxic effect on plant cells by inducing DON accumulation and probably enhances FHB susceptibility.

### 2.3. Phytohormone-Related Pathways Participate in the Regulation of F. graminearum Infection

#### 2.3.1. Jasmonic Acid and Ethylene Pathways

Plant defense in response to microbial attack is regulated through a complex network of signaling pathways that involve three signaling molecules including salicylic acid (SA), jasmonic acid (JA), and ethylene (ET) [42]. Both JA and ET pathways include two steps: the biosynthesis pathway and the signal pathway [43,44]. In wheat cultivar ‘Sumai3′, alleneoxide synthase, a key enzyme in JA biosynthesis was upregulated against *F. graminearum* inoculation, along with lipoxygenase enzyme, which indicated the induction of the JA pathway as a resistance mechanism [45]. The JA signal transduction pathway was normal in ‘Wangshuibai’ while it was blocked in the FHB-susceptible mutant ‘NAUH117′, which confirmed JA signaling was associated with FHB resistance [46]. The susceptibility of the ‘Meh0106’ mutant was caused partly by the failed timely positive regulation of the JA and ET pathway [47]. Moreover, ET and JA signaling may produce hydroxycinnamic acid amides to thicken the cell wall, resulting in resistance to FHB [48]. However, some reports hold that the ET pathway (ET biosynthesis/signal pathway) especially the ET signaling pathway does not confer FHB resistance, and the ET pathway might be unimportant for FHB resistance due to deactivation in ‘Wangshuibai’ while the ET pathway was not associated with, or might even compromise FHB resistance in wheat [46]. Arabidopsis mutants with reduced ET signaling were more resistant to *F. graminearum* than wild-type, while mutants with enhanced ET production were more susceptible, which demonstrated ET signaling is involved in FHB susceptibility [49]. In host resistance, the function of ET differs in the various interaction systems, improving resistance to some pathogens while increasing susceptibility towards others [50]. ET enhancers improved disease resistance of susceptible cultivars, but did not provide further protection for resistant genotypes, suggesting that the ET-signaling pathway may be naturally activated in resistant wheat, but not in susceptible wheat [51]. After reviewing the publications, we concluded that Type I resistance to FHB can be obtained from the synergistic effect between the ET and JA pathway, and the ET signal pathway alone may not be associated with FHB resistance in wheat. ET pathway-related resistance or susceptibility may vary from the various backgrounds of genotypes, eutrophic type of pathogen, and the timing activation order of other signal pathways.

#### 2.3.2. Salicylic Acid Pathway

Plants synthesizes salicylic acid (SA) via phenylalanine ammonia lyase (PAL) or gene isochorismate synthase 1 (ICS1) [52,53], whereas only PAL was responsive to *F. graminearum* infection [47], similar to another report that SA is synthesized via the PAL pathway in response to necrotrophic fungi *B. cinerea* in Arabidopsis plants [54]. SA signaling might be an early general defense response or part of the innate immune reaction to *F. graminearum* infection regardless of genotype, and it also acts as a prerequisite for later resistance development, while the SA synthesis deficiency mutant may result in its susceptibility [47]. Another report has shown that SA increased FHB resistance by reducing the germination and growth efficiency of *F. graminearum* at high concentrations [55]. The earlier activation of the SA pathway (SA biosynthesis/signal pathway) might be associated with FHB resistance while the delay in activation of this pathway may be related to FHB susceptibility in wheat. Activation of the JA signaling pathway followed activation of the SA defense pathway and plant defense signaling was suggested to occur in a sequential cascade, with SA signaling being active during the early phases of infection, followed by ET signaling and JA signaling in wheat [47].

#### 2.3.3. Abscisic Acid Pathway

Abscisic Acid (ABA) is one of the major plant hormones and plays an important role in plant immunity [56]. Although ABA can impact resistance either positively or negatively depending on the pathogen, the tide of evidence is leaning more toward ABA working as a susceptibility factor, at least with respect to fungal pathogens [56,57,58,59]. In the case of fungal responses, ABA has been shown to increase the susceptibility of Arabidopsis to *Fusarium oxysporum* [60,61]. What is more, exogenous application of ABA was shown to increase development of disease symptoms of *Magnaporthe oryzae* on barley [62]. Additionally, ABA influences defense against necrotrophs and biotrophs by enhancing JA signaling and suppressing SA signaling [63,64]. It is inconsistent with the previous conclusion that the JA signal pathway is involved in FHB resistance in wheat which might be due to the antagonistic effect between the JA and SA pathways. A recent transcriptome analysis presented that exogenous ABA application can dysregulate defense responses by further exacerbating gene expression and altering the cell wall strengthening mechanism, thus aggravating the occurrence of disease while gibberellins (GA) reduces FHB disease severity [65]. It seems that ABA is associated with FHB susceptibility and the SA signal pathway is associated with FHB resistance at the early infection stage.

The involvement of plant hormones and hormonal crosstalk in any plant–microbe interaction is complex [66]. The SA pathway is inhibited while the JA pathway is most likely stimulated during *F. graminearum* infection [35]. Another study also pointed out that plant resistance to biotrophic pathogens is classically thought to be mediated by the SA pathway, and by contrast, resistance to necrotrophic pathogens is controlled by the JA and ET pathways [67]. Although both SA and JA pathway function in FHB resistance, there seems to be an antagonistic effect between them. Future studies are obviously needed to understand the effect of plant hormones on cereal–*F. graminearum* interactions. This article will not be covering other phytohormone such as cytokinin (CTK) and indole-3-acetic acid (IAA), since there have been few reports on CTK and IAA in wheat–FHB interactions.

### 2.4. Pathogenesis-Related Protein in Host May Be Associated with Basal Defense Response

Pathogenesis-related (PR) proteins such as PR-1, PR-2 (β-1-3-glucanase), PR-3 (chitinase), PR-4 (wheatwin1), PR-5 (thaumatin-like protein), PR-9 (heme-containing glycoproteins), PR-10, PR-12 (defensin), PR-14 (Non-specific lipid transfer protein, LTP) have been shown to be induced or upregulated after pathogen infection and usually have higher abundance at an early stage (Table 1) [35,46,68,69,70]. NPR1 (non-expresser of PR-1) overexpression enhanced the resistance to FHB by sensitizing wheat plants to respond faster and stronger to SA [71]. LTPs (PR-14) have multiple roles in plant disease resistance. Higher abundance of LTP was reported to function in cutin deposition [72]. TaLTP3 was associated with the 5A QTL conferring Type I FHB resistance, exhibiting a high expression level in the resistant genotype [25]. Like TaLTP3, the overexpression of TaLTP5 in wheat confers increased FHB resistance [73]. In addition, PR-2 was transformed to susceptible wheat ‘Bobwhite’ to obtain transgenic plants with enhanced resistance to FHB [74]. In greenhouse experiments, transgenic wheat lines expressing PR-2 showed intensive resistance against *F. graminearum* [75] while the transgenic wheat line carrying a PR-2 did not show delayed susceptibility in field evaluations [74]. Similarly, transgenic wheat expressing a class II PR-3 that had a low level of PR-3 protein showed the highest level of resistance in the greenhouse but did not enhance resistance in the field trials [76]. The degree of improvement of resistance to FHB in PR transgenic wheat lines is still to be determined. PR proteins may be involved in Type I resistance to FHB because most PR proteins have higher abundance in resistant wheat genotypes than in susceptible wheat genotypes. In addition, transgenic wheat lines expressing some PR genes showed insignificant improvement of FHB resistance, indicating that single PR protein might be ineffective against FHB and the combination of several certain other PR proteins might have a significant effect on FHB resistance in wheat.

## 3. Type II Resistance

Type II resistance is the ability of wheat to resist the spread of FHB in the spike, which is estimated by delivering conidia into a single floret of a spike and calculating the percentage of symptomatic spikelets [8]. Infection hypha invaded host tissue by penetrating the inner layers of the host cell wall by 36 hpi, which occurs on the inner surfaces of lemma, glume, and palea, and on the upper part of the ovary [28], thereby the initial timepoint of hypha spread is 36 hpi and the host defense mechanism reached Type II resistance. Type II resistance has been extensively studied and widely utilized in breeding research, as its resistance is more stable and easier to assess [8].

### 3.1. QTL for Type II Resistance

The first QTL mapping paper published by Waldron et al. [77] detected five QTL for Type II resistance, and the QTL with the largest effect was derived from ‘Sumai3’ and was designated *Qfhs.ndsu3BS*. In recent years, hundreds of QTL associated with Type II resistance have been reported [9]. However, most of them showed minor effects and their expressions were greatly affected by the environment, genetic background, and inoculation method. Meta-QTL (MQTL) analysis of these QTL could shrink their confidence intervals and identify the QTL regions most commonly associated with trait variation [78,79,80]. A recent report conducted a meta-analysis of QTL for Type II and III resistances over the past 20 years, showing that a total of 529 QTL were for Type II resistance. The highest numbers of QTL were located on chromosomes 3B, 5A, and 2D, and the lowest numbers on chromosomes 1D and 6D [21]. Among all these reported QTL, *Fhb1* presents the largest effect on FHB Type II resistance and is more stable across multiple backgrounds [17].

### 3.2. Cell Wall Biomolecular Composition May Play an Essential Role in Resistance to FHB

Hemicellulose is the main component of plant cell wall and a kind of non-cellulose polysaccharide with a low degree of polymerization in plant tissues. The compositions which constitute hemicellulose are upregulated after pathogen infection in both resistant and susceptible wheat genotypes and these compositions may be involved in basal resistance to FHB. Hemicellulose in ‘Sumai3’ was stronger and more persistent, and remained uninterrupted after infection, but decreased in susceptible wheat cultivars, indicating that cell wall polymers may lead to an important factor in cell wall strength, thus achieving Type II FHB resistance [81]. Besides, the contents of arabinose, galactose, and glucose which composed hemicellulose were significantly higher in the resistant line ‘02- 5b-318R’ [82].

Hydroxyproline-rich glycoproteins (HRGPs) are the major components of the structural cell wall proteins which are involved in cell wall reinforcement at the site of pathogen infection [83]. Labelling densities of HRGP in cell walls of the *Fusarium culmorum* (*F. c*)-infected lemma, ovary, and rachis increased only slightly in the susceptible cultivar ‘Agent’ while increased markedly in the cell walls of infected tissues of the resistant cultivar ‘Arina’ compared with the healthy tissues [84].

Thionins are cysteine-rich polypeptides of about 5 kDa and are toxic to plant pathogens in vitro [85]. The expression of genes encoding protein thionins often increased as part of the plant host defense response to pathogen attack [86]. The content of thionine in the cell wall of susceptible wheat cultivar only increased slightly, which accumulated a lot in the host cell wall of resistant wheat cultivar after infection [84,87]. Additionally, expression of the gene encoding protein thionins in transgenic plants has been shown to enhance fungal resistance [88].

In general, most of the major structural proteins that make up the cell wall are likely to be involved in resistant response to FHB. ADF/cofilin proteins, proline-rich proteins (PRPs), and glycine-rich protein (GRP) were also upregulated after *F. graminearum* infection and differed in resistant and susceptible cultivars [46,89,90]. Notably, these components show higher expression in resistant wheat genotypes, particularly genotypes which carry the QTL *Fhb1*, proving the important role of cell wall components in Type II resistance.

### 3.3. Phenylpropanoid Pathway Enhances Resistance by Thickening Cell Wall

The phenylpropanoid pathway is one of the most important secondary metabolic pathways in plants, which give rise to metabolites including lignin, flavonoids, lignans, phenylpropanoid esters, hydroxycinnamic acid amides, and sporopollenin and play an important role in plant growth and development as well as plant–environment interaction [91]. Phenylalanine ammonia lyase (PAL), the first committed enzyme of the first dedicated step in the phenylpropanoid pathway, catalyzes the transformation of L-Phe to ammonia and trans-cinnamic acid, and acts as a substrate for the downstream phenylpropanoids, flavonoids, and lignan pathways [92]. PAL was significantly upregulated in spikelets of resistant barley genotypes following *F. graminearum* infection compared with mock/water inoculation and thus indicated the activation of the phenylpropanoid and flavonoid pathways [93]. PAL showed earlier induction and was significantly upregulated in *F. graminearum*-inoculated FHB resistant wheat genotypes ‘CM82036’ [94], ‘Sumai3’ [95], ‘Wangshuibai’ [47], and in the FHB-resistant line ‘GS-1-EM0040’ [70]. In addition, PAL was downregulated in the susceptible wheat genotype ‘Recital’ after *F. graminearum*-infection and in FHB-susceptible wheat ‘Superb’ after DON-inoculation [70]. These results suggested that PAL was induced or upregulated in resistant wheat genotypes, and its downregulation in susceptible wheat genotypes may be associated with FHB susceptibility. Notably, upregulation or downregulation of PAL first occurs before 36 hpi, indicating that it may play a role in Type I resistance, but the main function is to activate the phenylpropanoid pathway which plays a role in Type II resistance.

Phenylalanine (Phe) is the precursor of both the phenylpropanoid and flavonoid pathways. Phe was explicitly more abundant in wheat following DON treatment [96]. The concentration of Phe was found to be more increased in resistant barley genotypes compared to the susceptible ones following *F. graminearum* treatment [97]. Furthermore, Phe was identified as FHB Resistance Related Induced (RRI) metabolite in spikes of wheat NIL with resistant *Fhb1* allele [48].

Lignin is a complex racemic aromatic heteropolymer, which is derived from the methoxylation of hydroxycinnamyl alcohol monomer and deposited in the thickening process of the secondary cell wall [98]. Lignin is considered to improve plant disease resistance which aggregates at the site of pathogen infection to form a physical barrier to prevent the spread of pathogens [99]. A significant difference in lignin monolignols composition was detected between the resistant and susceptible plants [82]. Lignin accumulation was stronger and more persistent in the resistant ‘Sumai3’ compared to the susceptible cultivar [81]. Metabolome profiling detected a higher abundance of metabolites belonging to lignin biosynthetic pathways in wheat R-RIL than in S-RIL of QTL-*Fhb2* by 7 days upon *F. graminearum* inoculation [100]. Besides, the transcription of 1-aminocyclopropane-1-carboxylate (ACC) synthase, the major gene involved in lignin biosynthesis in the systemic defense-related group, was significantly highly expressed in highly-resistant genotype ‘Nobeokabouzu-komugi’ during *F. graminearum* infection [101]. What is more, genes described as laccase and blue copper protein, which were known as mediators of lignin polymerization [102,103], were strongly induced in all resistance groups after *F. graminearum* inoculation [104]. A recent study found that silencing of TaNAC032 transcription factor can significantly reduce the content of lignin in the rachis, thus increasing the susceptibility of transgenic wheat to *F. graminearum* infection [105]. Caffeoyl-CoA-O-methyltransferase (CCoAOMT) is the main regulator determining the efficiency of lignin synthesis and composition. The expression of CCoAOMT is induced in ‘Wangshuibai’ at an early stage of infection [47] which improves the efficiency of lignin synthesis and obtains Type II resistance. Lignin serves as a critical factor to strengthen the cell wall and accumulates to form a physical barrier to resist the penetration of the pathogen, both in resistant and susceptible genotypes.

Flavonoids are a large class of phenolic compounds with C_6_-C_3_-C_6_ structure based on 2-phenyl chromone. The precursors of its biosynthesis are phenylalanine and maronyl coenzyme A. Flavonoids can protect plant cell wall integrity upon fungal infection by inhibiting the activity of several plant cell wall degrading enzymes secreted by fungal pathogens to penetrate plant tissues [106]. Similar to lignin, flavonoids were induced only in NIL-R, or induced at higher levels in NIL-R than in the NIL-S [48,100]. Additionally, transgenic flax plants expressing higher levels of flavonoid glucosides exhibited increased resistance to *F. graminearum* infection [107]. The facts mentioned above indicated flavonoids are associated with FHB resistance in wheat.

Hydroxycinnamic acid amides (HCAAs) are considered to be one of the end products of the phenylpropanoid pathway, which are a family of conjugated complex induced metabolites, playing a dual role in pathogen resistance as phytoalexins and cell wall strengthening agents [108]. HCAAs were induced in higher abundance in ‘Sumai3’ compared to the susceptible cultivar Roblin [108]. A faster accumulation of HCAAs has also been reported in the *Fhb1* genotypes compared with the *Qfhs.ifa-5A* genotypes after DON treatment [96]. In addition, *TaACT* is a candidate gene related to FHB resistance in wheat QTL on 2DL, which reinforces the secondary cell wall by depositing HCAAs and prevents the further spread of pathogens throughout rachis [109]. Another study indicated that *TaWRKY70* gene elevates the accumulation of HCAA metabolites and mediates FHB resistance by regulating downstream *TaACT*, *TaDGK,* and *TaGLI* genes [110].

In short, most of the metabolites produced in the phenylpropanoid pathway are related to FHB resistance and play an essential role in Type II resistance by thickening the secondary cell wall or inhibiting the activity of the plant cell wall degrading enzymes (Table 2). Lignin provides plants with a mechanical support for cell walls to grow and transport water and nutrients throughout their life cycle [111]. Many key enzymes and genes in the process of lignin biosynthesis are upregulated after *F. graminearum* infection, indicating that lignin biosynthesis plays an indispensable role in Type II resistance to FHB.

## 4. Type III Resistance

FHB is caused primarily by *Fusarium graminearum* infection, leading to the accumulation of several mycotoxins in grain such as predominantly deoxynivalenol (DON), nivalenol (NIV), and zearalenone (ZEN) which make grain unsuitable for human consumption and livestock feed [112]. Low mycotoxin accumulation is Type III FHB resistance [4], which is also considered as a component of Type II resistance because it commonly reduces the spread of disease [113]. Boutigny et al. [114] further proposed classification of Type III resistance into two types: resistance to trichothecene accumulation by metabolic transformation of the toxin and resistance via inhibition of trichothecene biosynthesis. As mentioned above, 96 QTL for Type III resistance has been mapped by meta-QTL analysis. The 61 genetic map-based MQTL on chromosomes 2DS, 2DL, 3BS, and 5AL showed both Type II and Type III resistance to FHB [21]. It proves that Type II and Type III resistance are closely related and difficult to discuss separately. Therefore, we have mainly introduced genes or antimicrobial compounds related to detoxification and inhibition of DON biosynthesis in this review.

### Mycotoxin Detoxification

UDP-glucuronosyltransferases (UGTs) are one of the two major DON-responsive genes and they were potentially involved in DON detoxification [25]. Plants can chemically modify DON to produce DON-3-glucosides with less toxicity by UGTs [115]. It was suggested that wheat and barley can induce UGTs to respond to infection and detoxify the trichothecene mycotoxins [89]. UGT genes were expressed in both highly-resistant genotype and susceptible genotypes [89,94,101,115,116,117]. However, genes encoding UGTs were differentially expressed in NILs with resistant and susceptible alleles after infection [118,119] and had higher expression level in the FHB-resistant wheat genotypes ‘Dream’ and ‘Sumai3’ compared with the FHB-susceptible cultivar ‘Lynx’ [120]. Besides, transgenic wheat expressing barley *HvUGT1348* gene showed high levels of Type II resistance to a nivalenol (NIV) producing *F. graminearum* strain [121]. We consider UGTs are involved in basal FHB resistance which not only contributes to detoxification, but also participates in Type II resistance.

ATP-binding cassette transporters (ABC transporters) have been reported to detoxify the trichothecenes that are produced by *F. graminearum* through transportation of toxic compounds out of the cell [122]. ABC transporters are classified into distinct families based on their protein domain composition and organization [123]. Some of the best characterized subfamilies are pleiotropic drug-resistant (PDR)-like protein (ABCG), multidrug resistance protein (ABCB/MDR), and multidrug resistance-associated protein (ABCC/MRP) [123,124,125]. It has been suggested that wheat and barley can induce ABC transporters to respond to infection and mycotoxin detoxification [89]. For instance, *TaPDR1*, a member of the ABC protein superfamily, has been identified in cv. ‘Wangshuibai’ due to its strong upregulation upon DON treatment as well as *F. graminearum* inoculation [126]. Strong upregulation of genes encoding ABC transporters was revealed by RNA profiling of DON-treated barley cultivar ‘Morex’ spikes [127]. Moreover, high accumulation of transcripts of ABC transporters was exhibited in wheat genotypes with *Fhb1* QTL [46,48]. ABC transporter-4 (ABC4), belonging to several putative defence-associated genes, was located in the *Fhb2* QTL region and also increased Type II FHB resistance [11]. Furthermore, overexpression of an ABC transporter can enhance the resistance to trichothecenes in tobacco [70]. Pleiotropic drug-resistant (PDR)-like ABC transporters have been observed to be upregulated under conditions of environmental stress or the application of ABA [128]. Similar to UGTs, ABC transporters are crucial for FHB resistance which may be involved in different resistance types.

Glutathione S-transferases (GSTs) have been most intensively studied regarding herbicide detoxification in plants due to its important function in normal cellular metabolism and the detoxification of diverse xenobiotic compounds [129,130,131]. GST was markedly induced in NIL-R derived from ‘Sumai3’ and acted as a basal response to pathogen invasion [48]. Notably, TaGSTF5 was induced by FHB infection in ‘Ning7840’ but was not detected in *F. graminearum*-infected spikelets of ‘Clark’ [132]. GSTs exhibited higher transcriptional expression in DON-treated ‘Superb’ compared to water-treated control [70]. Recently, *Thinopyrum elongatum*–derived *Fhb7,* which encodes a GST that detoxifies trichothecenes, has been cloned [16]. In conclusion, GSTs may have higher levels in resistant wheat genotypes than susceptible genotypes and may be generally induced or upregulated in resistant wheat genotypes.

Cytochromes P450s (CYP450s) make up a component of the plant defense response to pathogens, wounding, and chemical treatments [133]. Genes encoding enzymes containing CYP450-like enzymes are involved in metabolism-related functions and CYP450s participated in the phenylpropanoid pathway, which has an important effect in the plant defense metabolism [134]. Particularly, CYP450s have two broad classes of activities containing biosynthetic pathways and detoxification pathways [134,135]. For example, trans-cinnamate 4-monooxygenase, a CYP450 involved in the pathway of lignin biosynthesis, catalyzes the oxidative reaction of trans-cinnamic acid to p-coumaric acid [136]. Several other studies have reported that CYP450s were expressed during FHB infection [69,133,137,138]. However, transcripts of one CYP450 gene accumulated only in resistant wheats and not in susceptible ones in response to *F. graminearum* compared to water controls [139]. Additionally, the CYP450 family comprises the largest class of enzymes in plants involved in the biosynthesis of plant hormones and metabolites, and has been shown to be induced in plants exposed to stress or pathogen infection [140]. Based on the observations above, we concluded that CYP450s not only participate in Type III resistance, but also contribute to Type II resistance by regulating lignin synthesis.

Other antimicrobial compounds such as magnolol, chitosan hydrochloride, and quinofumelin, etc. (Table 3) also inhibit the growth of *Fusarium* and production of DON. From the analysis of most reports, it is not difficult to perceive that most of the genes involved in detoxification are also related to Type II resistance, indicating that Type III resistance cannot be separated from Type II resistance. Moreover, it is worth noting that the general roles of these detoxification genes in different resistance types suggest that these genes can be utilized as candidate genes for the development of breeding new resistant cultivars.

## 5. Type IV and Type V Resistance

Type IV resistance was proposed by Mesterházy [5] but was not clearly defined. Researchers usually took damaged kernel rate (FDK) as the parameter for Type IV resistance. However, QTL for FDK mostly coincided with those for II resistance [9]. Recently, Gong et al. [6] recommended merging Type V into Type IV resistance because of their similarity in nature and suggested the reduction in thousand kernel weight (TKW) on the premise of same disease severity as an indicator for Type IV resistance, which simplified the FHB resistance types. Up to now, there have only been a few research works on omics for Type IV and V resistances. Perlikowski et al. [150] conducted a kernel proteomic analysis of two wheat lines differing in resistance levels and found that both monomeric alpha-amylase and dimeric alpha-amylase inhibitors were highly accumulated in the more resistant line after inoculation with *F. culmorum*, indicating that inhibition of pathogen alpha-amylase activity could be a crucial component of the resistance to FHB. In another research work [151], accumulation levels of plant beta-amylase were increased in a more susceptible triticale line after infection but not in resistant cultivars, suggesting that inhibition of pathogen alpha-amylase and plant beta-amylase activities may be associated with Type IV resistance to FHB.

## 6. Challenges and Prospectives

*F. graminearum* which causes FHB is thought to be a hemibiotrophic pathogen [152]. Any spike will be infected under a high pressure of *F. graminearum* conidia in the surrounding environment if suitable environmental conditions such as temperature and humidity encounter the wheat anthesis. Obviously, more work is needed to understand Type I resistance. Apart from this, although phytohormone and PR proteins have been induced or upregulated as early as 36 h after inoculation, some reports also indicated that transcriptional abundance of these may peak in the later stage [46,119], suggesting these pathways or proteins may play a more important role in Type II resistance compared to Type I resistance. Therefore, the mechanism of host-mediated Type I resistance to FHB and the corresponding phenotyping methods are still required to be carefully examined, or to be distinguished from Type II resistance.

Besides, we attributed contradictory reports on omics data, such as UGTs and ABC transporters, etc. [46,70], to host genetic backgrounds, *F. graminearum* strain/fungal biomass/necrotrophic stage or biotrophic stage, inoculation method/tissue/time/procedure, sampling method/tissue/time/procedure, plant growth stage, nutritional environment, the variability inherent in FHB research, data analysis platform/limited resolution of the technologies, size of the investigated RNA samples, and so on.

From the perspective of genomic level, the researches on FHB in recent decades has provided us with abundant information for genetic and molecular determinants, mainly focusing on the resistant genetic components. Recent reports demonstrated that the removal of the susceptibility interval on the short arm of chromosome 4D [153] and the deletion of *Sf-Fhb7AS* enhanced Type II resistance to FHB [154]. Therefore, in addition to finding the components or pathway related to disease resistance through the analysis of omics data, knocking out or knocking down the expression host susceptibility genes has provided new venues for protection against pathogens [155].

**Figure 1:**
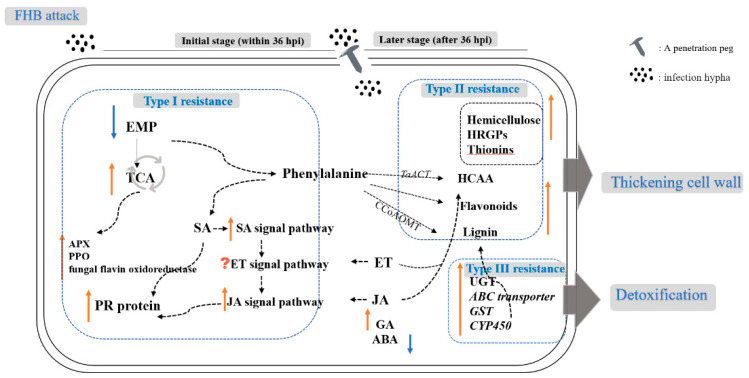
A model illustrating pathways and different types of FHB resistance. The dotted arrows represent indirect interactions; the vertical upward arrows represent a positive correlation with resistance and the vertical downward arrows represent a negative correlation with resistance. EMP, glycolysis pathway; TCA, tricarboxylic acid cycle; APX, ascorbate peroxidase; PPO, polyphenol oxidase; HRGPs, hydroxyproline-rich glycoproteins; HCAA, Hydroxycinnamic acid amide; UGT, UDP-glucuronosyltransferase; ABC transporter, ATP-binding cassette transporter; GST, glutathione S-transferase; CYP450, cytochromes P450.

Linking multi-omics to wheat resistance types provided us with new insights into the underlying mechanisms and relationships between different resistance types to FHB. Based on the publications, we propose a model (as shown in Figure 1) for the interaction between different components or pathways and response to FHB attack. Among these, we emphasize the specific accumulation of genes in TCA in Type I resistance, the important role of the phenylpropanoid pathway in cell wall strengthening and detoxification genes which function in different resistance types.

Despite contradictory publications on the part of omics data for host-*Fusarium* species complex, multi-omics, a multidisciplinary field, has a wide range of applications and is playing an increasingly significant role in breeding disease-resistance of various crops due to its rapid development. The continuous improvement of the data analysis platform, experimental procedure and method, sequencing technology, as well as cloning and transgenic techniques would greatly promote the application of multi-omics, which will help decipher the complicated mechanism of different resistance types underlying FHB in wheat at the integral level. Based on the differential responses of the host to different types of resistance, we could make corresponding improvements in breeding work in the future.

## Figures and Tables

**Table 1:** Pathogenesis-related proteins responsive to *F. graminearum* infection.

PR Proteins	Properties	*F. graminearum* Infection	Abundance
PR-1	Unknown	induced	R > S ^1^
PR-2	β-1-3-glucanase	induced	R > S
PR-3	Type I, II, IV, V, VI, VII chitinase	induced	R > S
PR-4	Type I, II chitinase	induced	R > S
PR-5	Thaumatin-like protein	induced	R > S
PR-9	Peroxidase	induced	R > S
PR-10	Acidic proteins	induced	R > S
PR-12	Defensin	induced	R > S
PR-14	Non-specific lipid transfer protein	induced	R > S

^1^ The abundance of PR proteins in resistant cultivars was higher than in susceptible cultivars.

**Table 2:** Metabolites or enzymes in the phenylpropanoid pathway responsive to *F. graminearum* infection.

Phenylpropanoid Pathway	Function	*F. graminearum* Infection	Abundance
Phenylalanine ammonia lyase	catalyzes the transformation of L-Phe	upregulated in R ^1^	/
		downregulation in S ^2^	
Phenylalanine	precursor of phenylpropanoid pathway	induced	R > S ^3^
Lignin	strengthens cell wall	induced	R > S
Flavonoid	inhibits the activity of cell wall degrading enzymes	induced	R > S
Hydroxycinnamic acid amides	strengthens cell wall	induced	R > S

^1^ resistant cultivars; ^2^ susceptible cultivars; ^3^ the abundance of metabolites or enzymes in phenylpropanoid pathway in resistant cultivars was higher than susceptible cultivars.

**Table 3:** Antimicrobial compounds inhibit the growth of *Fusarium* and production of DON.

Antimicrobial Compounds	Properties	Reference
Magnolol	hydroxylated biphenyl-type neolignans	[141]
Quinofumelin	a novel quinoline fungicide	[142]
Thymol	a natural plant-derived compound	[143]
Epoxiconazole	demethylation inhibitor (DMI)	[144]
Fengycin	produced by *B.amyloliquefaciens* FZB42	[145]
Validamycin A	aminoglycoside antibiotic	[146]
Succinat dehydrogenase inhibitors	a class of fungicides that act on succinate dehydrogenase	[37]
Chitosan Hydrochloride	chitosan hydrochloride	[147]
Selenomethionine (SeMet)	converted by inorganic Se	[148]
Ethylenediaminetetraacetic acid disodium salt (EDTANa2)	a chelating agent targeting divalent cations	[149]
